# Increase of catastrophic health expenditure while it does not have socio-economic anymore; finding from a district on Tehran after recent extensive health sector reform

**DOI:** 10.1186/s12913-019-4418-1

**Published:** 2019-08-14

**Authors:** Mohammad Hassan Kazemi-galougahi, Elham Dadgar, Zahra Kavosi, Reza Majdzadeh

**Affiliations:** 1 Department of Epidemiology & Biostatistics, School of Public Health, Tehran University of Medical Sciences, Tehran, Iran; 2 Department of Health Management and Economics, School of Public Health, Tehran University of Medical Sciences, Tehran, Iran; 3 Department of Health Care Management, School of Management and Medical Informatics, Shiraz University of Medical Sciences, Shiraz, Iran; 4 Community Based Participatory Research (CBPR) Center, Tehran University of Medical Sciences, #1547, North Kargar St, Tehran, Iran

**Keywords:** Catastrophic health expenditures, Inequality, Equity, Iran

## Abstract

**Background:**

Ensuring financial protection of the community against health care expenditures is one of the fundamental goals of the health system. Catastrophic health expenditures (CHE) occurs when out-of-pocket health expenditures due to health care expenses considerably affect family life. The main purpose of this study was to analyze CHE trend over time and to determine its determinants.

**Methods:**

The last round of a three part study over time was conducted in June to September 2015 on 600 households in a non-affluent area of Tehran. The World Health Survey questionnaire was used to collect information. Health expenditure was considered to be catastrophic when OOP health expenditures exceed 40% of household’s capacity to paysubsistence expenditures. After calculating the amount of households’ exposure to CHE, determinants resulting in CHE using logistic regression and the amount of economic inequality in the exposure of households to CHE using the concentration index were calculated. Then, performing a decomposition analysis, the contribution of each of the studied variables to the observed economic inequality was determined. All the findings were compared with the results of studies carried out in the years 2003 and 2008.

**Results:**

In the year 2015, 29.9% of households incurred CHE. This amount was 12.6 and 11.8% in the 2003 and 2008 studies, respectively. The concentration index was - 0.017(confidence interval; − 0.086 to 0.051), which, unlike the CI calculated in the years 2003 and 2008, was not significant. The most important determinant affecting the exposure to CHE was inpatient service utilization (OR = 1.64).

**Conclusion:**

Comparing to the whole national wide findings in sum, in 2015, the amount of the exposure of the studied households to CHE was significant, and it in comparison with the results of the previous studies was increased. However, there was no significant economic inequality and the observed levels of inequalityin comparison with the results of the previous studies conducted in 2003 and 2008 were decreased.

## Background

One of the main goals of health systems is ensuring financial protection of the community against health care expenses. Direct payment is the most unfair and inefficient way of paying in the health system and can make households exposed to catastrophic health expenditures (CHE) [20, 27, 32].

Exposure to CHE occurs when a large part of the household’s available income is spent on out-of-pocket (OOP) payments for health services and thus leads the household to poverty. According to the World Health Organization, health expenditure is considered to be catastrophic when OOP health expenditures exceed 40% (in some studies, 10, 20, 25%, or 40% of total expenditure) of household’s capacity to paysubsistence expenditures (i.e., available income after fixing basic needs) [21, 34, 36, 37]. This happens more often in low-income and middle-income countries, where most of their health costs are paid out of their pockets.

Several regional studies, which have been conducted in this area in Iran, have estimated the exposure of families to CHE to be between 8.3 and 22.2% [23]. A systematic review and meta-analysis on 27 cross-sectional studies, estimated the pooled prevalence of CHE in Iran during 1995 and 2015 to be 3.91% (95% confidence interval, CI: − 3.26- 11.07) [7]. In another study based on Statistical Center of Iran (SCI) Surveydata, the exposure rate to CHE in rural and urban households in 2015 to 2016 estimated 5.65 and 4.58%, respectively [17].

Since households living in low-income areas are more likely to be exposed to CHE, its regular monitoring in such areas is highly important. In studies conducted in 2003 and 2008 on households in district 17 of Tehran, which was one of low-income areas in Tehran, the households’ exposure to CHE was estimated to be 12.6 and 11.8%, respectively [11].

Another important goal of health systems, in addition to improving health, is health equality through its proper distribution among different people with different economy levels [20]. Thus besides, the percentage of the exposure to CHE, what matters to us is the level of inequality in CHE and the determinants affecting it. In the studies conducted in 2003 and 2008 on households in District 17 of Tehran, the concentration indices of facing CHE were - 0.17 and - 0.19, respectively.

According to the census conducted in 2016, the 17th district of Tehran had 252,913 inhabitants and 81,530 households. This region was considered as a low-income region of Tehran and was located in the south of the capital with an area of 822.09 ha.

One of the advantages of study on a small defined population is to enable us comparing CHE variations and its inequality in different periods and investigate its trend. The present study was the third round in which the data were collected at specific intervals in 2015 (during the months of June to September), while the same previous studies were conducted in 2003 and 2008 [11].

What made this study even more important than other ones was the matter of the implementation of the Health Transformation Plan (HTP) in 2014 as one of the most important health-related events in Iran over the past three decades. Comparison of households exposed to CHE during the years before and after the implementation of HTP can help us to evaluate the access to the main goals of this plan.

The ultimate goals of HTP consisted of increasing the responsiveness of the health system, reducing financial risk due to health expenses. The first phase of the HTP was implemented in the governmental hospitals affiliated with Ministry of Health and Medical Education (MoHME) from May 5, 2014.One of the parts of this phase was the reduction in the cost of hospitalization of patients qualified for basic health insurance by 6% of the total hospitalization expenditures for urban households and 3% of the total hospitalization expenditures for rural households and residents of cities with less than 20 thousand population. The second phase of the plan, which began on May 22, 2014, focused on the primary healthcare. In the third phase of the plan, eliminating informal payments received by the medical community was taken into account. Since unrealistic medical tariffs were reported to be one of the most important reasons for asking for informal payments, the government decided to increase medical tariffs and make them closer to the real final prices. Thus, the book of relative value units of health services on September 29, 2014, was published with the aim of increasing tariffs and eliminating informal payments and establishing equity in the income of diverse specialties [16, 23].

The purposes of the present study were to determine the exposure of households in a low-income area of Tehran (17th district) to CHE, the inequality in households’ exposure to CHE, and the determinants affecting this inequality. In addition, the findings of the 2015 study (after HTP implementation) were compared with those of the 2003 and 2008 studies (before HTP implementation), and the differences were scrutinized.

## Methods

### Study design

In 2003, the research center in Tehran conducted a study on a representative sample of households in the area to identify the health needs of these households and to conduct the necessary interventions to meet these needs. In the study carried out in 2008, the same households were re-examined, and in 2015 we also looked at the same households and compared the changes [11] and households that could not be included in the study for any reason were replaced with neighboring households.

### Sampling and sample size

In 2003 survey, cluster sampling was used based on geographically identified building blocks in 17th district of Tehran. According to geographical sampling frame established by Statistical Centre of Iran, 64 clusters (i.e. building blocks) were selected by systematic sampling so that each building block consisted up to 18 households. Some of these 18 households were randomly selected to fill out the questionnaire. The selected sample included 1123 households, from which expenditure data were collected completely at random for 635 households. Finally 579 households (91.2%) with appropriate data selected for analysis [10, 24, 26].

In 2008, it was also attempted to study the households who were sampled in the 2003 survey. Of the 603 precise addresses available, 27 households were replaced with neighboring households using simple random sampling approach within the household’s living place (to choose neighboring households a matching approach was considered for total expenditures and size of households) for various reasons and 11 households’ data were incomplete, so finally the data provided by 592 households were analyzed [11].

**Fig. 1:**
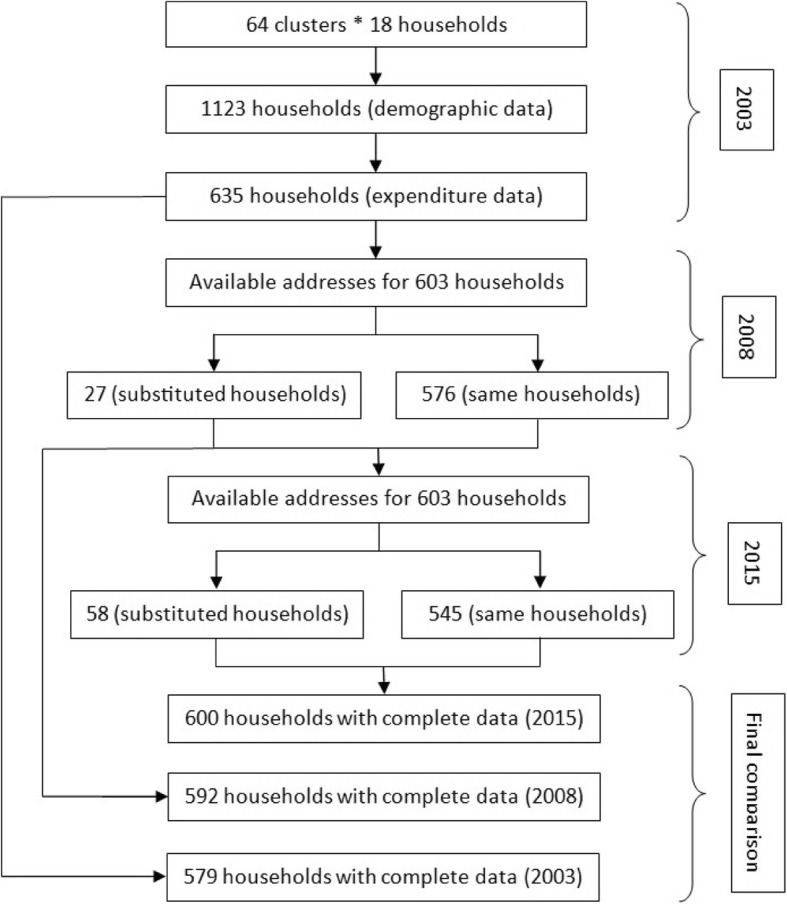
Sampling design of the studies conducted in 2003, 2008, and 2015

In 2015, we did our best to have the sample of the same households (or addresses) in 2008. Of the 603 precise addresses available from the 2008 survey, 58 households were replaced with neighboring households for various reasons (including non-residential addresses, building demolition, changed street plates, and households’ unwillingness to respond), and eventually data provided by 600 households were complete to be analyzed (Fig. 1).

### Data collection

The World Health Survey (WHS) questionnaire developed by the World Health Organization in order to monitor health system performance of different countries was used in the present study. Its reliability and validity were also confirmed [30].

The WHS included two main sections: the household questionnaire and the individual questionnaire. The household questionnaire included the roster of all the individuals in the household, household health intervention coverage, health insurance, health expenditure and indicators of permanent income. The individual questionnaire included sociodemographic features, health state description, health state evaluation, risk factors, mortality, coverage of health services and health system responsiveness [30].

The household questionnaire was answered by one of the over 18-year-old households who was able and willing to answer the questions and had the most information about general household information, household expenditure, and households’ usage of health care services. Then, the selected respondent was selected using Kish tables and responded to the second part of the questionnaire (i.e., the individual questionnaire) [4].

If the interview could not be carried out with the selected respondent because of addresses which were not accessible due to building demolition, households who were not willing to participate after calling them up to 5 times, or any other reasons, the participants were replaced by the right-neighboring households.

In this study, quality assurance was performed by arranging a detailed operational instruction for the interviewers and holding two training sessions for the interviewers to fully justify them before starting to collect data. During the data collection phase, quality control was conducted by telephoning 10% of the households and checking the collected data.

In 2003, a cluster sampling method was used for sampling. Sixty-four clusters were identified by a systematic sampling of the geographic sampling frame prepared by the Statistical Centre of Iran. Each of these clusters included 18 households who were randomly selected to answer the questionnaire [11].

This study was approved by the Ethics Committee of Tehran University of Medical Sciences. All the studied participants read and signed the informed consent forms. The collected information provided by the households was also completely confidential. In order to motivate the selected households to cooperate more and increase the response rate, a small gift, which included a health package containing a tube of toothpaste and two toothbrushes, was given to households before the inquiry was started.

### Statistical analysis

In this study, the households’ exposure to CHE, the determinants resulting in CHE, and the inequality in the exposure of households to CHE were calculated. Then, using the decomposition analysis method, the contribution of each of the studied variables to the observed inequality was determined and compared with the results of the 2003and 2008 rounds of the study.

Households with health expenditures over 40% of their capacity to pay were categorized under those households exposed to CHE. At first, equivalent household size was obtained from the actual household size raised to the power of the constant β value (equivalent to 0.56). In order to get the equivalent household food expenditures, we divided household food expenditures by the equivalent household size.

Then, food expenditure ratio to total household expenditures was calculated. The average equivalent food expenditure of households whose food expenditure ratio to total household expenditure was in the 45th to 55th percentile range was considered as the poverty line. Then, householdsubsistence expenditure was calculated based on WHO proposed method [37].

The household’s capacity to pay was obtained by the following equation:

1 $$ \mathbf{Household}'\mathbf{s}\ \mathbf{capacity}\ \mathbf{to}\ \mathbf{pay}\ \left({\mathbf{CTP}}_{\mathbf{i}}\right)=\mathbf{The}\ {\mathbf{i}}^{\mathbf{th}}\ \mathbf{household}'\mathbf{s}\ \mathbf{expenditure}\ \left({\mathbf{EXP}}_{\mathbf{i}}\right)-\mathbf{Subsistence}\ \mathbf{expenditure}\ \mathbf{of}\ \mathbf{the}\ {\mathbf{i}}^{\mathbf{th}}\ \mathbf{household}\ \left({\mathbf{SE}}_{\mathbf{i}}\right) $$

If household subsistence expenditure was higher than household food expenditure, then in the above formula household food expenditure would be used instead of subsistence expenditure.Ultimately, households with health expenditures more than 40% of their capacity to pay were categorized under those households exposed to CHE [1, 5, 12, 14, 28, 36]. It should be noted that beside the threshold level of 40%, we calculated households exposed to CHE at threshold level of 10, 20 and 25% of the total household expenditure.

Two poverty lines were used for measuring OOP relatedimpoverishment: 1. Household subsistence expenditure (equivalent household size multiplied by the average equivalent food expenditure of households whose food expenditure ratio to total household expenditure were in the 45th to 55th percentile range); and 2. Global poverty line developed and used by the World Bank (1.90 USD per day) multiplied by equivalent household size. The households were impoverished due to OOP if their total household expenditure minus health expenditures be below the poverty line. It should be noted that we excluded households who their total expenditure were below the poverty line before considering health expenditures.

A binary logistic regression was used to find out the key determinants affecting CHE. In this mode CHE was considered a outcome and economic status, lack of insurance, female household head, having member with age higher than 65 years old, having member with age lower than 5 years old, having disabled member, dentistry services usage and inpatient&outpatient service usage were considered as the predictors in our model.

2 $$ \ln \mathrm{odds}\ {y}_i=\alpha +{\sum}_k{\beta}_k{X}_{ki}+{\epsilon}_i $$

where *y*_*i*_ is exposure to CHE by household *i*, β is parameter, x_*i*_ is determinant and ε_i_ is the disturbance term.

In realizing the key determinants affecting CHE, we used the OECD-modified equivalence scale in order to adjust a household’s income based on its size and composition. In this method, a weight of 1.0 was assigned for the household head, a weight of 0.5 was assigned for other adult households, and a weight of 0.3 was assigned for members of households less than 15 years. In order to eliminate the effect of the number and composition of household members as a result of calculations, the household expenditure was divided by total calculated weights [2].

In order to determine socioeconomic inequality in CHE, the concentration index and concentration curve were used. The concentration index is actually defined as twice the area between the concentration curve and the line of equality (or the 45-degree line) and its values lie between +1 and -1. The negative values of the concentration index indicate the exposure concentration to CHE among the low-income households and the positive values of the concentration index indicate the exposure concentration to CHE among the high-income households [13, 33]. Finally, the findings of the 2015 study were compared with the results of the same studies done in the years 2003 and 2008.

Decomposition analysis was used to determine the contribution of each of the studied variables to the inequality in CHE, as described by Wagstaff et al. [18, 19, 31, 35]. The decomposition of the concentration index was based on the regression analysis of the relationship between the exposure to CHE and the variables studied.

The concentration index was used to measure socioeconomic inequality in CHE:

3 $$ C=\frac{2}{\mu }{\mathit{\operatorname{cov}}}_w\left({y}_i,{R}_i\right) $$

where y_i_ and R_i_ are the CHE status of the *i*th household and the fractional rank of the *i*th household in terms of the index of household expenditure respectively; μ is the mean of CHE in sample and *cov*_w_ denotes the weighted covariance.

Then, we regress CHE, *y*_*i*_ on dependent variables:

4 $$ \ln \mathrm{odds}\ {y}_i=\alpha +{\sum}_k{\beta}_k{X}_{ki}+{\epsilon}_i $$

where *y*_*i*_ is exposure to CHE by household *i*, β is parameter and ε_i_ is the disturbance term.

The concentration index for *y*_*i*_ can be weitten as:

5 $$ C={\sum}_k\frac{\beta_k{\overline{x}}_k}{\mu }{C}_k+\frac{G{C}_{\varepsilon }}{\mu }={C}_{\hat{y}}+\frac{G{C}_{\varepsilon }}{\mu } $$

where μ is the mean of y, ${\overline{x}}_k$ is the mean of x_k_, C_k_ is the concentration index for x_k_, and, *GC*_*ε*_ is the generalized concentration index for ε_i_ as residual.

The first component of Eq. (5) is equal to weighted sum of the concentration indices of the regressors and called “elasticity”. An elasticity is the change percentage in the dependent.

variable (exposure to CHE) associated with a change percentage in the explanatory variable.

The absolute contribution of each determinant can be calculated by multiplying the CHE elasticity with respect to that determinant and its concentration index. The contribution percentage of each determinant can be calculated through dividing its absolute contribution by the concentration index of the CHE [18, 19, 31, 35].

Eventually, the results of the 2015 study were compared with those of the 2008 study. It should be pointed out that decomposition analysis was not carried out in the 2003 study.

After the analysis was finished, since estimated CHE in 2015 was over our expectation, the analytical steps were repeated again as part of the quality control procedures.

## Results

The mean household size was 3.5 ± 1.5.The food-related poverty line in the population studied was 3,041,887 Rials (1 USD in 2015 ≈ 35,000 Iranian Rials). The mean of household expenditure on health services in 2015 was 10.5 times more than the calculated mean in 2003, and it was 3.9 times more than the calculated mean in 2008.Some characteristics of households are shown in Table 1.

**Table 1:** Characteristics of the studied population in 2003, 2008, and 2015

Variables	2003	2008	2015
Mean of total monthly household expenditure in Iranian Rials (Equivalent USD at the time of the study)	3,063,955 (~US$340)	3,835,511 (~US$426)	14,164,700 (~US$405)
Mean of total monthly household expenditure on health services in Iranian Rials (Equivalent USD at the time of the study)	250,801 (~US$28)	672,848 (~US$75)	2,637,262 (~US$75)
% households with disabled member	N/A	17.4	11
% households with female head	N/A	12.4	13
% households having member over 65 years	13	20	27.7
% households having member under 5 years	22	18	11.7
% households having health insurance	78	74	84.8
Household size	4.2	3.9	3.5

In 2015, 9.8% of households fell below the poverty line due to health expenditures; thus, this amount was decreased compared to the results of the 2003 study, and increased compared tothe results of the 2008 study (Table 2).

**Table 2:** Frequency of impoverished households due to health expenditures in the years 2003, 2008, and 2015

Impoverishment	2003		2008		2015	
	Frequency	Percent (confidence interval)	Frequency	Percent (confidence interval)	Frequency	Percent (confidence interval)
Have	48	10.2 (7.7–13.4)	31	5.5 (3.7–7.6)	53	9.8 (8.2–13.9)
Not Have	419	89.8 (86.6–92.3)	538	94.5 (92.4–96.3)	437	90.2 (86.1–91.8)
Total	467	100	569	100	490	100

When considering the global poverty line used by the World Bank (1.90 USD per day), 3.1% of households were impoverished households due to health expenditures in 2015.

In order to investigate the relationship between the studied variables and the exposure of households to impoverishment, a multivariate analysis of logistic regression was used. The results are presented in Table 3.

**Table 3:** Associations between the key determinants and impoverishment in 2015

Variables	B	SE	*P*-value	Adjusted OR 2015	95% confidence interval	
					Lower	Upper
Economic status				*		
Quintile 1 (poorest)	1.083	1.240	0.382	2.954	0.260	33.545
Quintile 2	1.843	1.101	0.094	6.317	0.730	54.695
Quintile 3	3.909	1.055	< 0.001	49.854	6.309	393.938
Quintile 4	4.669	1.076	< 0.001	106.580	12.942	877.735
Lack of Insurance	−0.612	0.513	0.233	0.542	0.198	1.483
Female household head	0.120	0.526	0.819	1.128	0.402	3.165
Having member ≥65 in Household	0.016	0.413	0.969	1.016	0.452	2.284
Having member ≤5 in Household	0.217	0.576	0.707	1.242	0.401	3.845
Household size						
3–6 members	−.499	0.496	0.314	0.607	0.230	1.604
≥ 7 members	−1.899	1.164	0.103	0.150	0.015	1.466
Having disabled member in household	0.088	0.586	0.880	1.092	0.346	3.466
Dentistry service usage	0.395	0.356	0.267	1.484	0.739	2.980
Inpatient service usage	0.582	0.398	0.144	1.789	0.819	3.906
Outpatient service usage	0.558	0.673	0.407	1.746	0.467	6.531
Constant	−5.389	1.244	< 0.001	0.005*		

*P < 0.05

If the threshold level of OOP health expenditures was considered 10, 20 and 25% of the total household expenditure, 62.7, 51.5 and 46.6% of the households had been exposed to CHE in 2015, respectively.

**Fig. 2:**
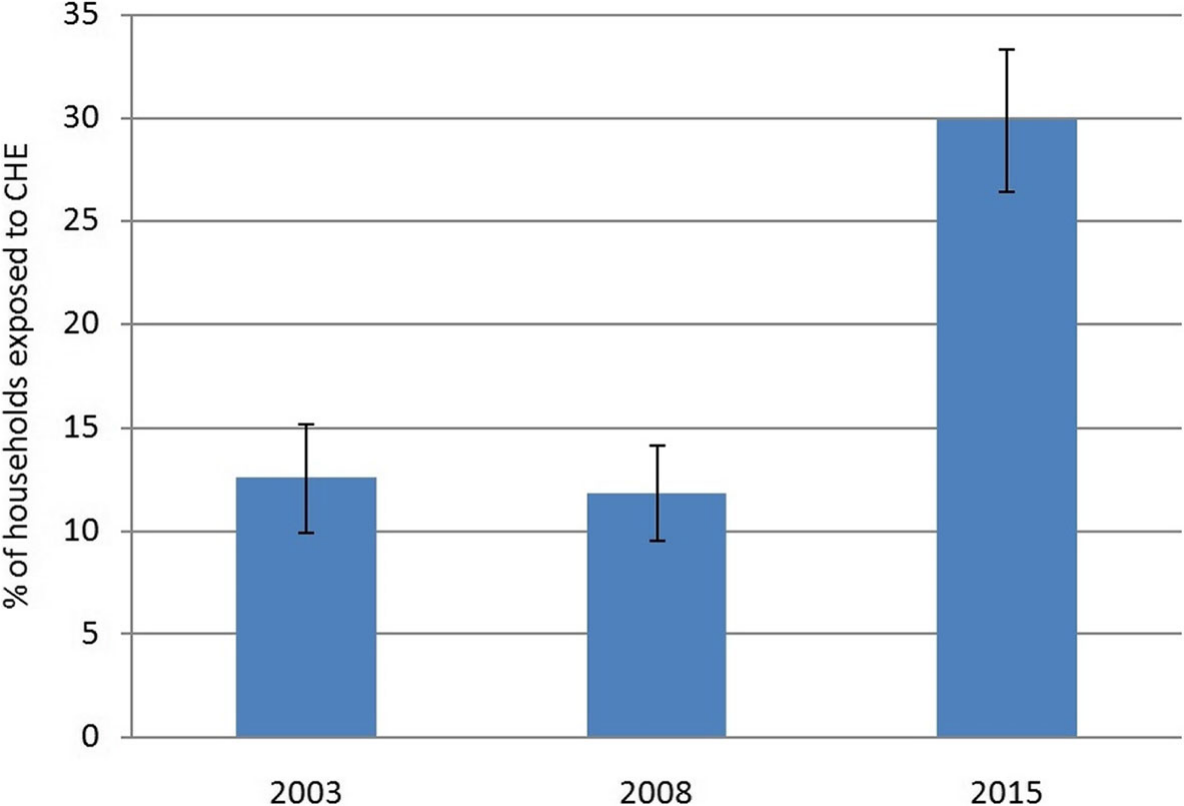
Percentages of households exposed to CHE in 2003, 2008, and 2015

At the threshold level of 40%, 29.9% of the households surveyed in 2015 had been exposed to CHE; this amount was increased up to 2.4 times compared to the results of the 2003 study, and up to 2.5 times compared to the results of the 2008 study (Fig. 2).

**Fig. 3:**
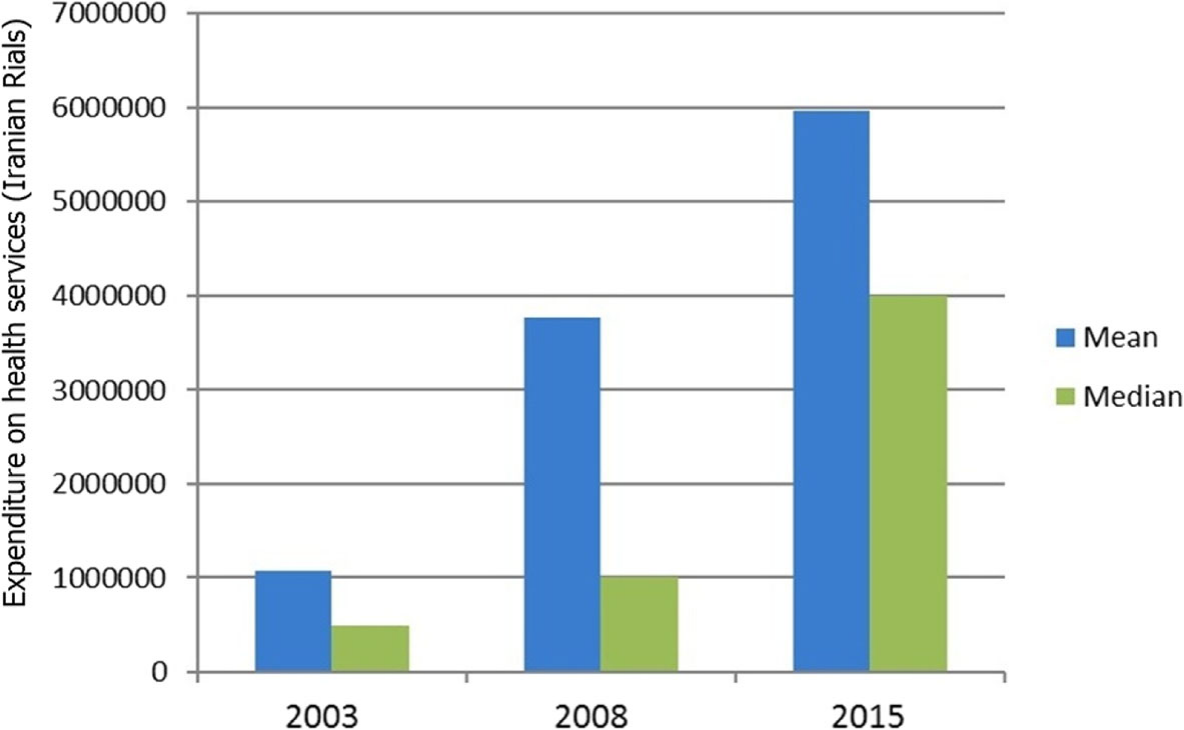
Comparison of the mean and the median expenditure on health services for households exposed to CHE in the years 2003, 2008 and 2015 (Iranian Rials)

The median expenditure on health services for households exposed to CHE in 2015 was six times more than that of the 2003 study and four times more than that of the 2008 study (Fig. 3).

The characteristics of households exposed to CHE in 2003, 2008, and 2015 are depicted in Table 4.

**Table 4:** Number and proportion of households exposed to CHE by study variables in the 2003, 2008, and 2015 surveys

Variables	2003 survey N (%, CI)	2008 survey N (%, CI)	2015 survey N (%, CI)	*P*-value^a^
Total no. households with CHE	73 (12.6%, 9.9–15.3)	70 (11.8%, 9.2–14.4)	179 (29.9%, 26.2–33.6)	< 0.001
Economic status		*		
Quintile 1 (poorest)	25 (17%, 10.9–23.1)	23 (18%, 11.3–24.7)	38 (35.3%, 26.2–44.4)	< 0.001
Quintile 2	16 (14%, 7.6–20.4)	9 (14%, 5.5–22.5)	32 (34.8%, 25.1–44.5)	< 0.001
Quintile 3	15 (14%, 7.4–20.6)	21 (13%, 7.8–18.2)	48 (32%, 24.5–39.5)	< 0.001
Quintile 4	9 (9%, 3.4–14.6)	8 (9%, 3.1–14.9)	22 (25.3%, 16.2–34.4)	0.001
Quintile 5 (richest)	8 (7%, 2.3–11.7)	9 (6%, 2.2–9.8)	39 (32.5%, 24.1–40.9)	< 0.001
Insurance status			*	
Have	52 (11%, 8.2–13.8)	47 (11%, 8.0–14.0)	160 (33.3%, 29.1–37.5)	< 0.001
Not have	21 (17%, 10.4–23.6)	23 (15%, 9.3–20.7)	19 (22%, 13.2–30.8)	0.382
Household head				
Father	–	62 (11%, 8.4–13.6)	150 (30.5%, 26.4–34.6)	< 0.001
Mother or other	–	8 (18%, 6.6–29.3)	29 (39%, 27.9–50.1)	0.017
Member ≥65 years in Household		*		
Have	14 (11%, 5.6–16.4)	15 (14%, 7.4–20.6)	56 (35.6%, 28.1–43.1)	< 0.001
Not have	59 (12%, 9.1–14.9)	55 (11%, 8.3–13.7)	123 (30.1%, 26.6–34.5)	< 0.001
Household size				
1–2 members	18 (19%, 11.1–26.9)	3 (12%, −0.7-24.7)	54 (38.5%, 30.4–46.7)	< 0.001
3–6 members	48 (11%, 8.1–13.9)	48 (10.5%, 7.7–13.3)	120 (29.6%, 25.1–34.1)	< 0.001
≥ 7 members	7 (12%, 3.6–20.4)	19 (17%, 10.0–23.0)	5 (23.8%, 5.6–42.0)	0.434
Disabled member in the household		*		
Have	–	21 (20%, 12.3–27.6)	22 (35.9%, 23.9–47.9)	0.023
Not have	–	49 (10%, 7.3–12.7)	157 (31.1%, 27.1–35.1)	< 0.001
Dentistry usage (during previou**s** month)	**	**		
Have	27 (27%, 18.3–35.7)	29 (14%, 9.3–18.7)	75 (35.1%, 28.7–41.5)	< 0.001
Not have	46 (10%, 7.3–12.7)	41 (8%, 5.6–10.3)	104 (29.6%, 24.8–34.4)	< 0.001
Inpatient service usage (during previous year)	*	**	*	
Have	12 (30%, 15.8–44.2)	13 (56.5%, 36.2–76.8)	51 (42.1%, 33.3–50.9)	0.114
Not have	61 (12%, 9.2–14.8)	57 (10%, 7.5–12.5)	128 (28.8%, 24.6–33.0)	< 0.001
Outpatient service usage (during previous month)		**	*	
Have	–	67 (14.5%, 11.3–17.7)	170 (32.9%, 28.8–36.9)	< 0.001
Not have	–	3 (2%, −0.2-4.2)	9 (18.5%, 7.6–29.4)	< 0.001

*P < 0.05; **P < 0.001

^a^Chi-square test

In order to investigate the relationship between the studied variables and the exposure of households to CHE, a multivariate analysis of logistic regression was used. The results are presented in Table 5.

**Table 5:** Prediction of key determinants of CHE in 2003, 2008, and 2015

Variables	Adjuster OR 2003	95% confidence interval	Adjuster OR 2008	95% confidence interval	Adjuster OR 2015	95% confidence interval
Economic status			*			
Quintile 1 (poorest)	3.10	(1.26–7.79)	5.03	(1.82–13.87)	1.47	(0.77–2.80)
Quintile 2	2.55	(0.99–6.51)	4.19	(1.13–13.76)	1.55	(0.81–2.95)
Quintile 3	2.19	(0.85–5.62)	2.78	(1.06–7.25)	1.31	(0.73–2.33)
Quintile 4	1.44	(0.51–4.05)	1.96	(0.64–6.99)	0.65	(0.33–1.28)
Lack of Insurance	1.93*	(1.05–3.51)	2.42*	(1.26–4.65)	0.62	(0.33–1.18)
Female household head	–	–	1.79	(0.69–4.69)	1.46	(0.81–2.63)
Having member ≥65 in Household	1.66	(0.79–3.48)	1.43	(0.69–2.95)	1.14	(0.70–1.84)
Having member ≤5 in Household	0.82	(0.41–1.64)	1.13	(0.53–2.49)	1.30	(0.67–2.53)
Household size						
3–6 members	0.62	(0.31–1.22)	0.62	(0.15–2.53)	0.60	(0.36–1.01)
≥ 7 members	0.93	(0.32–2.62)	1.13	(0.24–5.38)	0.34	(0.10–1.16)
Having disabled member in household	–	–	1.31	(0.64–2.63)	0.78	(0.40–1.51)
Dentistry service usage	4.09**	(2.31–7.24)	4.58**	(2.36–8.91)	1.39	(0.92–2.10)
Inpatient service usage	3.52**	(1.61–7.69)	11.39**	(3.76–34.57)	1.64*	(1.02–2.62)
Outpatient service usage	–	–	1.51**	(1.29–1.77)	1.003	(0.998–1.007)

*P < 0.05; **P < 0.001

**Fig. 4:**
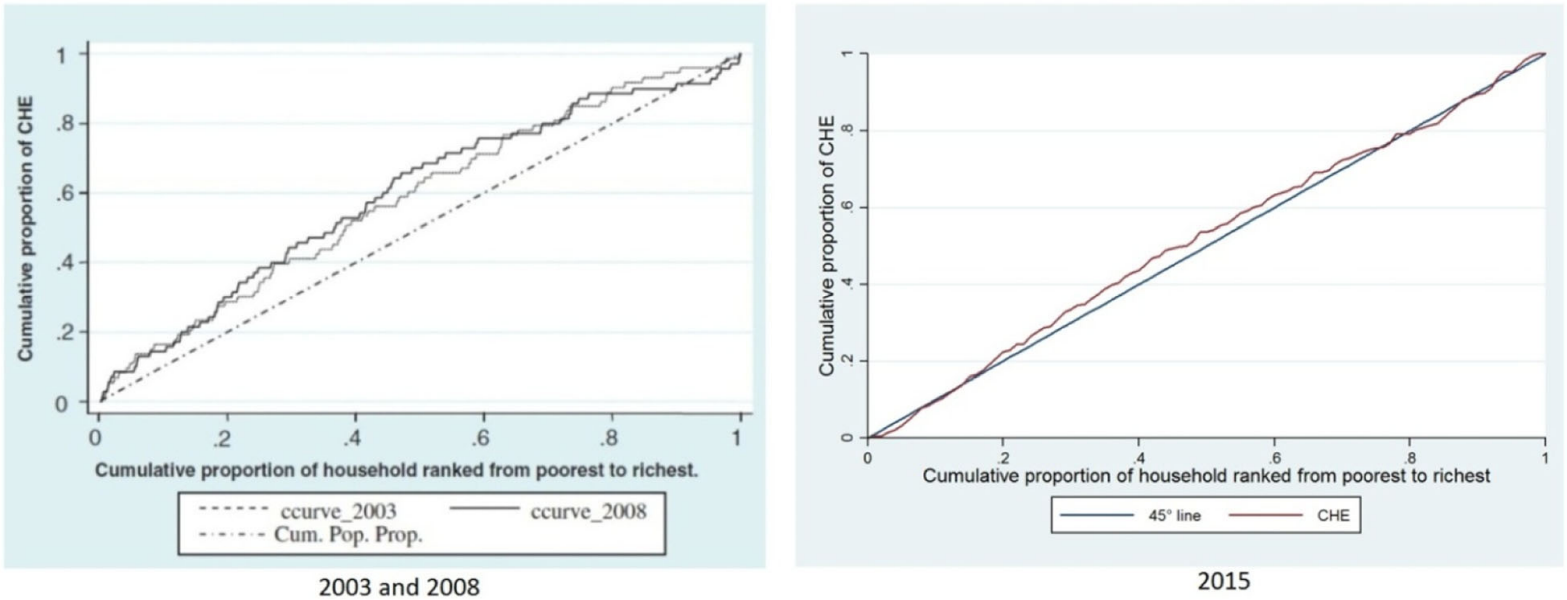
Concentration curves of income-related inequality in facing CHE in 2003, 2008, and 2015

The inequality of exposure to healthcare expenditures in 2015 was as pro-poor as in the 2003 and 2008 studies, but it declined compared with the previous studies. Moreover, unlike the previous twostudies, it was not significant (Table 6 and Fig. 4).

**Table 6:** Concentration indices of facing CHE in 2003, 2008, and 2015

Year of study	Concentration index	Confidence interval	
		Upper limit	Lower limit
2003	0.17-	0.3-	0.04-
2008	0.19-	0.06-	0.32-
2015	0.017-	0.086-	0.051

Decomposition analysis was applied to determine the role of each of the variables under consideration in dealing with CHE, and its results were compared with the results of the 2008 study (Table 7).

**Table 7:** Decomposition analysis of concentration index of CHE in 2008 and 2015

Variables	Coefficient		Mean		Elasticity		Concentration index (CI)		Contribution to CI %	
	2008	2015	2008	2015	2008	2015	2008	2015	2008	2015
Economic status									83%	220.2%
Quintile 1 (poorest)	1.402	−0.385	0.216	0.194	−0.115	−0.055	−0.785	0.823		
Quintile 2	1.282	− 0.820	0.108	0.167	− 0.053	− 0.086	− 0.46	0.466		
Quintile 3	1.026	−0.117	0.280	0.269	−0.109	− 0.021	− 0.071	−0.059		
Quintile 4	0.689	0.052	0.152	0.157	−0.039	0.006	0.362	−0.461		
Lack of Insurance	0.836	0.472	0.263	0.848	−0.084	0.268	−0.095	0.031	6%	−21.4%
Female household head	0.573	0.378	0.076	0.130	−0.016	0.033	−0.389	0.102	5%	−8.5%
Having member ≥65 in Household	0.567	0.129	0.204	0.277	− 0.044	0.024	− 0.099	−0.059	3%	3.6%
Having member ≤5 in Household	0.374	0.261	0.179	0.117	−0.025	0.020	−0.068	0.032	1%	−1.6%
Household size									8%	−75.1%
3–6 members	−0.604	0.560	0.772	0.713	0.177	0.268	0.069	−0.080		
≥ 7 members	−0.078	1.073	0.186	0.037	0.005	0.180	−0.357	0.284		
Having disabled member in household	0.335	−0.254	0.174	0.110	− 0.022	−0.019	−0.357	0.022	2%	1.1%
Dentistry service usage	1.589	0.329	0.154	0.372	−0.092	0.082	0.102	0.038	−7%	−7.9%
Inpatient service usage	2.497	0.493	0.039	0.212	−0.036	0.070	−0.032	0.013	1%	−2.3%
Outpatient service usage	1.789	0.003	0.782	32.40	−0.531	0.060	0.005	0.106	−2%	−16.1%

## Discussion

One of the main indicators of development in a society is the degree of health equity in its population, how fairly health is distributed across the social spectrum [15]. The financial protection of community against health care expenses is one of the main goals of health systems. Direct payment can make households exposed to CHEwhich indicates the existence of health inequity in the people of that society [36].

A systematic review and meta-analysis estimated the pooled prevalence of CHE in Iran during 1995 and 2015 to be 3.91% [7]. In another study the exposure rate to CHE in urban households in 2015 to 2016 estimated 4.58%, [17]. Despite relatively low rate of CHE exposure in national studies, we calculated it as 29.9%in 17th district of Tehran in 2015. It should be noted that although the calculated CHEexposure rate in this district was higher than country’s, it may be due to different characteristics of this region compared with the whole country: low-socioeconomic status, high immigrants rate and work of many residents in hard physical occupations can be some factors explaining these differences.

According to the results of the present study, the percentage of households exposed to CHE in 2015 had a significant increase compared with the results of the surveys conducted in 2003 and 2008, in which it was 12.6 and 11.8%, respectively.

It seems that the following six main determinants can be effective in making these changes:

1. ***The implementation of the HTP:***

This plan is considered as one of the most important health-related events in Iran over the past three decades. The ultimate goals of the HTP contain increasing the responsiveness of the health system, reducing OOP payments, reducing the exposure percentage of households to CHE, and increasing the amount of child natural delivery. One part of the first phase of the HTP was the reduction in the hospitalization expenditures of patients qualified for basic health insurance by 6% of the total hospitalization expenditures for urban households and 3% of the total hospitalization expenditures for rural households and residents of cities with less than 20 thousand population. In the third phase of the plan, eliminating informal payments received by the medical community was taken into account. Since unrealistic medical tariffs were reported to be one of the most important reasons for asking for informal payments, the government decided to increase medical tariffs and make them closer to the real final prices. Thus, the book of relative value units of health services on September 29, 2014, was published with the aim of increasing tariffs and eliminating informal payments and establishing equity in the income of diverse specialties [16, 23].

After the implementation of the HTP, the proportion of OOP payments was reduced, but some experts believed that the sharp increase in public health tariffs did not decrease the final OOP payments, and even in some cases there was an increase.

2. ***The implementation of the Iranian targeted subsidy plan:***

This plan has been implementing in Iran from December 18, 2010.According to it, during the 5 years of subsidy, the subsidy cuts on 16 items of goods and services, including gasoline, gas oil, gas, oil, electricity, water, wheat, sugar, rice, cooking oil, and milk and they are all sold at an international price. According to the plan, the government earned 2000 trillion Iranian Rials for this rise in price, and it was planned to distribute half of that income among people in exchange for counteracting inflation. Although in the early years of the plan, cash subsidies were paid to all Iranian households (a monthly rate of 445,000 Rials, approximately equivalent to 13 dollars, per each member of the household), gradually the government began to de-register the more affluent households from the beneficiaries’ list [8, 9, 29].

What may have led to the presence of an information bias in the current study is that the study carried out in 2015, contrary to the 2003 and 2008 studies, followed the implementation of the targeted subsidy plan. Based on the training provided to the interviewees, the research objectives of this study, the confidentiality of the economic information received, the non-relevance of this study to the subject of subsidies and taxes, and so on were completely described for the households under study. Despite all the anticipated measures, given that the study was conducted in a relatively poor area of Tehran, small cash subsidies for many of these households were very important. Moreover, the respondents enjoyed lower levels of education than those in other districts of Tehran. Moreover, these people may be less likely to respond accurately to questions and may report their own income level less than the actual amount and express their health expenditures more than the real amount, which could be a factor causing an information bias in the study and lead to the overestimation of the households’ exposure to CHE.

3. ***Inflation:***

The annual inflation rate in the Iran’s economy has led to an increase in the prices of goods and services and, consequently, an increase in health costs. The average annual inflation rate between 2003 and 2015 was 18.5% [3, 6, 22]. According to the results of this study, the mean expenditures of household health services in 2015 was 10.5 times more than that of the 2003 study, and it was 3.9 times more than that of the 2008 study. Increasing healthcare expenditures can be one of the most important factors in increasing households’ exposure to CHE.

4. ***The difference between causes of exposure to CHE in poor and rich households:***

Another point that should not be overlooked is that the amount of utilization of health services among high-socioeconomic status (SES) households is different from that of low-SES households; thus, households’ exposure to CHE among households with different SES occurs due to different reasons. For example, in a household with a favorable economic status, performing a cosmetic surgery may result in the exposure to CHE, but in low-income households, basic health interventions may also lead to the exposure to CHE.

5. ***Change in diagnostic and medical procedures and subsequent health expenditures:***

Diagnostic and medical procedures are changing over time thus subsequent health expenditures are not comparable in 2015 with 2003 and 2008; for example, MRI prescription was not so common in 2003 and 2008 in Iran while it is highly used in recent years. Increase in households exposed to CHE in 2015 can be relevant to this issue.

6. ***Change in health care utilization:***

Increase in health care utilization is one of the main causes of CHE exposure. Despite we measured the health care utilization in 2015, we could not compare them since data of 2003 and 2008 were not available. It is probable that one of the causes of growth in CHE exposure in 2015 is the increase in health care utilization compared to 2003 and 2008.

Furthermore, regarding the inequality in households’ exposure to CHE in 2015 compared to that of the 2003 and 2008 studies, the inequality in all the three studies was pro-poor and inequality in low-income households was more concentrated, but there was a significant decline in inequality in 2015 as compared to previous studies. In other words, it can be concluded that in the study done in 2015, compared to the 2003 and 2008 studies, the proportion of households exposed to CHE was increased more equitably.

It should also be noted that all studied households had been selected from a low-income area of Tehran and economic similarity of these households may lead to reduction in CHE inequity.

In decomposition analysis, as in 2008, among the studied variables, the largest share was related to economic status (although the share of this variable in 2015 was higher than that of the 2008 study). However, the direction of the effect of the variables such as lack of health insurance, being a female head of household, the presence of a member under 5 in the household, the size of the household, and the usage of hospitalization services due to inequality in households’ exposure to CHE was reversed, but the direction of the effect of the variables including having a member over 65 years in the household, having a disabled member, dentistry service usage, and inpatient service usage was in line with that of the study conducted in 2008 (Table 7). One of the most important limitations of current study is about the poorest segments of the population which do not use any healthcare services, and hence they won’t even appear in the analysis of determinants of CHE. The standard logit approach outlined in this study does not take such considerations into account. The medical care seeking behavior of households should ideally be accounted for in order to accurately assess the risk factors associated with incurring catastrophic health expenditure. In order to control for the potential sample selection issue associated with the fact that households can only incur catastrophic health care expenditure if they actually seek and purchase health care, we follow the approach proposed by Sartori [25].

## Conclusion

To put it into a nutshell, it can be concluded from this study that in the 2015 survey compared to 2003 and 2008 ones, the proportion of households exposed to CHE had a significant increase, but at the same time, equality amongst households was increased.

Conducting panel studies on these 600 households at a future time can be helpful in estimating the exposure to CHE in urban households located in less affluent areas and increasing our understanding of the inequality status in these households.

## Data Availability

The datasets generated during and analysed during the current study are available from the corresponding author on reasonable request.

## Abbreviations

CHE: Catastrophic health expenditures
CI: Concentration index
CTP: Capacity to pay
EXP: Expenditure
HTTP: Health Transformation Plan
MoHME: Ministry of Health and Medical Education
OOP: Out-of-pocket
SE: Subsistence expenditure
SES: Socioeconomic status
VAT: Value added tax
WHS: World health survey
