# Correction to: Increase of catastrophic health expenditure while it does not have socio-economic anymore; finding from a district on Tehran after recent extensive health sector reform

**DOI:** 10.1186/s12913-019-4542-y

**Published:** 2019-10-16

**Authors:** Mohammad Hassan Kazemi-galougahi, Elham Dadgar, Zahra Kavosi, Reza Majdzadeh

**Affiliations:** 1 Department of Epidemiology & Biostatistics, School of Public Health, Tehran University of Medical Sciences, Tehran, Iran; 2 Department of Health Management and Economics, School of Public Health, Tehran University of Medical Sciences, Tehran, Iran; 3 Department of Health Care Management, School of Management and Medical Informatics, Shiraz University of Medical Sciences, Shiraz, Iran; 4 Community Based Participatory Research (CBPR) Center, Tehran University of Medical Sciences, #1547, North Kargar St, Tehran, Iran

**Correction to: BMC Health Serv Res (2019) 19:569**

**https://doi.org/10.1186/s12913-019-4418-1**

In the original publication of this article [1], the title should be revised as blow:

Increase of catastrophic health expenditure while it does not have socio-economic inequality anymore; findings from a district in Tehran after recent extensive health sector reform.

## References

[1] Kazemi-galougahi MH (2019). Increase of catastrophic health expenditure while it does not have socio-economic anymore; finding from a district on Tehran after recent extensive health sector reform. BMC Health Serv Res.

